# Editorial: Mechanisms and models of musculoskeletal pain and nonpharmacological treatment

**DOI:** 10.3389/fnint.2022.998413

**Published:** 2022-08-10

**Authors:** William R. Reed, Kenneth A. Weber, Daniel F. Martins

**Affiliations:** ^1^Department of Physical Therapy, University of Alabama at Birmingham, Birmingham, AL, United States; ^2^Division of Pain Medicine, Stanford University School of Medicine, Palo Alto, CA, United States; ^3^Experimental Neuroscience Laboratory (LaNEx), Postgraduate Program in Health Sciences, University of Southern Santa Catarina - UNISUL, Palhoça, Brazil

**Keywords:** musculoskeletal, pain, non-pharmacological, manual therapy, integrative medicine, non-pharmaceutical, animal model

This Research Topic is an introduction to much needed mechanistic-oriented research that can eventually inform and/or optimize non-pharmacological clinical interventions for functional improvement and pain management. This collection of articles acknowledges the complexities involved in performing mechanistic-oriented non-pharmacological research in both clinical and preclinical studies. For instance, a persuasive argument concerning application of forces using hands vs. machines is presented by Bove et al. who identify important experimental variables that need to be considered such as size, shape, and density differences of various human/machine force applicators, as well as tissue biomechanical differences based on tissue density and viscoelastic behavior (i.e., muscle, fat, and bone) in designing mechanistic-oriented manual therapy studies (Bove et al.). Three articles in this topic address central/peripheral inflammatory cytokines or immunoregulatory effects of either joint mobilization or preventative omega-3 supplementation in various preclinical pain models. Omura et al. demonstrated that ankle joint mobilization reduces nociceptive behavior, pro-inflammatory cytokines IL-1β and TNF in central (spinal cord) tissues, while not altering peripheral (hind paw) tissue cytokine levels in a complete Freund adjuvant induced pain model. Joint mobilization failed to reduce hind paw M1 macrophage infiltration, but did decrease M2 macrophage density after tissue inflammation (Omura et al.). Demonstrating central and peripheral neuroimmunomodulatory effects of manually applied peripheral joint mobilization is an important step in identifying potential physiological mechanisms of decreased pain/nociception subsequent to non-pharmacological manual therapy treatment. Preventive omega-3 supplementation produced antihyperalgesic and anti-inflammatory effects in part by reducing pro-inflammatory IL-1β (peripheral) and TNF (central) cytokine concentrations (Galassi et al.). TGF-β1, TNF, and MCP-1 cytokine concentrations were not altered, however IL-10 concentrations were reduced in hind paw muscle but not in skin tissue, as well as the prevention of an injury-induced reduction of anti-inflammatory IL-4 concentrations peripherally (Fernandes et al.). Using an established rat model of forelimb repetitive strain injury (i.e., an overuse injury model) that induces sensorimotor declines, broad neuromuscular inflammation and tissue fibrosis, Barbe et al. found that massage therapy enhanced muscle anti-inflammatory IL-10 concentrations even after the repetitive injury had become fully established. Manual therapy combined with rest elicited modest improvement over rest alone in metrics indictive of neuropathy such as forepaw/forelimb agility, cold sensitivity, slow median nerve conduction velocity and neural fibrosis in repetitive strain injury animals. These results contrast with the robust preventative effects of massage therapy demonstrated previously using this same model (Barbe et al.). Combined, these preclinical studies provide support for preventative and/or early non-pharmacological interventions in minimizing early effects of pro-inflammatory response at both the peripheral site of tissue injury as well as centrally. Additional research is needed to inform longer term effects and whether the transition from acute to chronic pain can be reduced or prevented using these and/or other non-pharmacological interventions. In the sole clinical study of this Research Topic, Gevers-Montono et al. investigated changes in TNF-α urine concentrations in relation to multimodal chiropractic care (comprised primarily of spinal manipulation). Individuals with chronic low back pain had significantly higher urine TNF-α levels compared to control levels, and chiropractic care resulted in a significant reduction in TNF-α levels accompanied by lower levels of pain and disability. Limitations of this preliminary study include a small sample size, non-standardized number and duration of treatments, and lack of a sham treatment group. Therefore, caution must be exercised in interpretating this early study measuring changes in urine pro-inflammatory cytokines associated with non-pharmacological treatment.

In the only cadaveric study of this Research Topic (Funabashi et al.) provided a novel perspective of spinal manipulative force distribution within spinal tissues. Force distributions within spinal tissues were determined during simulated spinal manipulation (30 N preload, 300 N peak force, time to peak 112.5 ms and consequent 2.6 N/ms loading rate) delivered by a servo-controlled linear actuator to the skin overlying L3/L4 in fresh porcine cadavers. Results indicated that only a small percentage of applied spinal manipulation forces actually reach spinal structures (median peak force of 36.4 N of the 300 N applied; equivalent to 12.1%). Of the forces reaching the spinal structures, over 96% are experienced by the intervertebral disc which is comparable to intradiscal pressures reported during daily functional activities such as walking, sitting combined with flexion, and even lower than intradiscal pressures during sit to stand movement or lifting 20 kg. Supraspinous and interspinous ligaments experienced 0.5% of mean forces, while the facet joints and ligamentum flavum experienced 3% of mean forces. Clarifying the proportion of applied spinal manipulative forces reaching deep spinal tissues along with their respective distribution will help advance our understanding of physiological mechanisms underlying this popular form of non-pharmacological treatment for spinal pain, while potentially helping to improve its clinical efficacy and safety.

The final article in this Research Topic was a systematic review and activation likelihood estimation (ALE) meta-analysis on sensorimotor integration and perception (Gombaut and Holmes). While most of nociceptive-related research has focused on primary pain receptors and/or afferent cortical activity distribution, the impact of efferent motor processes on sensory processing has received growing interest of late as the motor system is becoming recognized to play important roles in pain management. The primary motor cortex is extensively interconnected with descending pain modulatory regions and sensory processes of the brain. This ALE-meta-analysis article showed that the supplementary motor area has greater activity in persons with atypical afferent activity in persons with *no pain* vs. persons *in pain*, as well as above-normal efferent motor activity being associated with decreased activity in brain regions involved in affective processing. Additionally, this article provides support for four hypotheses offering additional insights into the impact of motor processes on central brain regions implicated in nociceptive processing. Looking at the other side of sensory processing offers a potentially important non-pharmacological source of analgesia, physical, and mental health.

While mechanistic-oriented non-pharmacological research remains in developmental stages as a field of research, public interest and clinical utilization of these interventions continues to increase year over year as evidenced by nearly 30,000 views of this Research Topic in the past 8 months.

## Author contributions

WR wrote the first draft. KW and DM provided critical comments and editorial suggestions for revisions. All authors agreed on the submitted version.

## Conflict of interest

The authors declare that the research was conducted in the absence of any commercial or financial relationships that could be construed as a potential conflict of interest.

## Publisher's note

All claims expressed in this article are solely those of the authors and do not necessarily represent those of their affiliated organizations, or those of the publisher, the editors and the reviewers. Any product that may be evaluated in this article, or claim that may be made by its manufacturer, is not guaranteed or endorsed by the publisher.

